# Phosphoproteomic Alterations of Ionotropic Glutamate Receptors in the Hippocampus of the Ts65Dn Mouse Model of Down Syndrome

**DOI:** 10.3389/fnmol.2018.00226

**Published:** 2018-07-25

**Authors:** Macarena Gómez de Salazar, Cristina Grau, Francisco Ciruela, Xavier Altafaj

**Affiliations:** Neuropharmacology Unit, Bellvitge Biomedical Research Institute (IDIBELL)—University of Barcelona, Barcelona, Spain

**Keywords:** Down syndrome, ionotropic glutamate receptors, NMDA receptor, kinases, proteomics, phosphoproteomics

## Abstract

Down syndrome (DS), the main genetic cause of intellectual disability, is associated with an imbalance of excitatory/inhibitory neurotransmitter systems. The phenotypic assessment and pharmacotherapy interventions in DS murine models strongly pointed out glutamatergic neurotransmission alterations (specially affecting ionotropic glutamate receptors [iGluRs]) that might contribute to DS pathophysiology, which is in agreement with DS condition. iGluRs play a critical role in fast-mediated excitatory transmission, a process underlying synaptic plasticity. Neuronal plasticity is biochemically modulated by post-translational modifications, allowing rapid and reversible adaptation of synaptic strength. Among these modifications, phosphorylation/dephosphorylation processes strongly dictate iGluR protein–protein interactions, cell surface trafficking, and subsynaptic mobility. Hence, we hypothesized that dysregulation of phosphorylation/dephosphorylation balance might affect neuronal function, which in turn could contribute to the glutamatergic neurotransmitter alterations observed in DS. To address this point, we biochemically purified subsynaptic hippocampal fractions from adult Ts65Dn mice, a trisomic mouse model recapitulating DS phenotypic alterations. Proteomic analysis showed significant alterations of the molecular composition of subsynaptic compartments of hippocampal trisomic neurons. Further, we characterized iGluR phosphopattern in the hippocampal glutamatergic synapse of trisomic mice. Phosphoenrichment-coupled mass spectrometry analysis revealed specific subsynaptic- and trisomy-associated iGluR phosphorylation signature, concomitant with differential subsynaptic kinase and phosphatase composition of Ts65Dn hippocampal subsynaptic compartments. Furthermore, biochemical data were used to build up a genotype-kinome-iGluR phosphopattern matrix in the different subsynaptic compartments. Overall, our results provide a precise profile of iGluR phosphopattern alterations in the glutamatergic synapse of the Ts65Dn mouse model and support their contribution to DS-associated synaptopathy. The alteration of iGluR phosphoresidues in Ts65Dn hippocampi, together with the kinase/phosphatase signature, identifies potential novel therapeutic targets for the treatment of glutamatergic dysfunctions in DS.

## Introduction

Down syndrome (DS) is the most common genetic cause of intellectual disability (OMIM #190685). Individuals with DS present a trisomy of the human chromosome 21 (Hsa21), and pivotal genes located in Hsa21 have been proposed as candidate genes for DS pathophysiology (Antonarakis et al., 2004). To model DS pathophysiology, different animal models have been used, either overexpressing single Hsa21-homologous genes or encompassing a partial trisomy of Hsa21 syntenic region (Dierssen, 2012; Rueda et al., 2012). Among the latter, Ts65Dn mouse is the most well-established DS murine model and genetically consists of the triplication of the Hsa21 syntenic mouse chromosome Mmu16. Ts65Dn mouse recapitulates cognitive and motor alterations observed in individuals with DS (Costa et al., 1999; Galdzicki and Siarey, 2003; Altafaj et al., 2013). These alterations are neurochemically associated with an imbalance of excitatory/inhibitory neurotransmitter systems (Belichenko et al., 2009), similar to those described in individuals with DS (Risser et al., 1997; Seidl et al., 2001). This neurotransmitter imbalance resulting from the alteration of GABAergic inhibitory neurotransmission (Fernandez et al., 2009; Best et al., 2012; Contestabile et al., 2017), together with excitatory glutamatergic neurotransmission defects (Siddiqui et al., 2008; Kleschevnikov, 2012), might disturb synaptic plasticity and impair learning and memory abilities in Ts65Dn mice. Consequently, several attempts have been made toward the restoration of homeostatic excitatory/inhibitory balance. In particular, Ts65Dn treatment with memantine—a reversible *N*-methyl-d-aspartate glutamate receptor (NMDAR) antagonist—has been shown to partially rescue critical electrophysiological alterations (Scott-McKean and Costa, 2011) and cognitive phenotypic abnormalities of trisomic mice (Costa et al., 2007; Lockrow et al., 2011). These findings have been significantly translated to the clinical practice, with an improvement of the cognitive skills of individuals with DS (Boada et al., 2012). These data strongly support the hypothesis that Hsa21 gene overexpression (e.g., DYRK1A, APP, TIAM1, and ITSN1 gene products) might dysregulate NMDAR expression and/or function, contributing to DS-like pathogenesis in trisomic mice (Siddiqui et al., 2008). NMDARs belong to the ionotropic glutamate receptor (iGluR) family, together with α-amino-3-hydroxy-5-methylisoxazole-4-propionic acid receptors (AMPARs) and kainate receptors (KARs). iGluRs importantly play a critical role in fast-mediated transmission, a process underlying synaptic plasticity and neuronal survival (Paoletti et al., 2013). The precise control of iGluR activity is tuned by numerous post-translational mechanisms (Wang et al., 2006; Traynelis et al., 2010). Among them, (de)phosphorylation events strongly influence iGluR biogenesis (assembly, trafficking, docking, surface diffusion, and internalization) and receptor channel gating. These processes modulate synaptic strength and thus modulate neuronal plasticity (Lee, 2006; Lau and Zukin, 2007; Goebel-Goody et al., 2009; Pahl et al., 2014). The phosphorylation pattern of iGluR results from the activity of several canonical protein kinases (PKA, PKC, CaMKII) (reviewed by Lussier et al., 2015) and protein phosphatases (STEP, PP2A, PP1, calcineurin) (Westphal, 1999; Chan and Sucher, 2001; Braithwaite et al., 2006; Sanderson et al., 2016) that collectively modulate the gating and subcellular location of iGluR. It has been proposed that the specific subsynaptic location of NMDARs (synaptic vs. extrasynaptic) plays a dichotomic role related with synaptic plasticity and neuronal survival (Hardingham and Bading, 2010). We have recently showed that the gene product of DS candidate gene *DYRK1A* (dual-specificity tyrosine phosphorylation-regulated kinase 1) also influences NMDAR surface expression and function similar to NMDAR regulation by Hsa21 gene products (Grau et al., 2014). To sum up, these data suggest that the overexpression of Hsa21 genes impacts glutamatergic transmission in DS. This potential functional disturbance might molecularly be associated with and/or result from a complex reorganization of the synaptic proteome composition and protein phosphopattern, with protein-specific dysregulation (subsets of proteins under-represented, over-represented, with conserved expression and with protein and site-specific phosphorylation changes). To identify these potential molecular changes, in this study, we have optimized a biochemical subfractioning method allowing the purification of subsynaptic compartments (pre-, extra-, and postsynaptic fractions) for further proteomic and phosphoproteomic analysis of Ts65Dn adult mice hippocampi. Phosphoenrichment-coupled mass spectrometry analysis revealed a subsynaptic-specific and disease-associated iGluR phosphopattern signature. This phosphopattern is in agreement with the concomitant subsynaptic-specific increase of kinase expression and phosphatase downregulation in Ts65Dn mice hippocampi. Overall, our results demonstrate an altered phosphopattern in the glutamatergic synapse, together with the identification of a protein kinase/phosphatase biochemical signature in the Ts65Dn murine model, which may represent novel therapeutic targets for DS synaptopathy.

## Methods

### Mice

Ts65Dn mouse colony female B6EiC3Sn a/A-Ts(1716)65Dn (Ts65Dn) and male B6C3F1/J mice were purchased from the Jackson Laboratory (Bar Harbor, ME). The mouse colony was housed and bred in the Animal Facilities of the Barcelona Biomedical Research Park (PRBB, Barcelona, Spain, EU). All animal procedures met the guidelines of the European Community Directive 86/609/EEC and were approved by the Local Ethics Committee. The mice were housed under a 12:12 h light–dark schedule (lights on at 8:00 a.m.) in controlled environmental conditions of humidity (60%) and temperature (22 ± 2°C) with food and water *ad libitum*. Both Ts65Dn and euploid mice were genotyped by qPCR, following the Jackson laboratories protocol (https://www.jax.org/research-and-faculty/tools/cytogenetic-and-down-syndrome-models-resource/protocols/cytogenic-qpcr-protocol).

### Antibodies

Antibodies were obtained from commercial suppliers as it follows: anti-PSD95 (Neuromab, #P78352), anti-synaptophysin (Sigma-Aldrich, S5768), anti-β-actin (Sigma-Aldrich, A5316), anti-GluN2A (Sigma-Aldrich, M264), anti-GluN1 (Millipore, #AB9864R), anti-CaMKIIα (CST, #50049), anti-Fyn (CST, #4023), anti-pS890 GluN1 (CST, #3381), anti-pS1284 GluN2B (CST, #5355), anti-rabbit IgG-horseradish peroxidase (HRP; Dako, #P0448), and anti-mouse IgG-HRP (Dako, # P0447).

### Subsynaptic fractionation and western blot

Subsynaptic fractionation protocol was adapted from the protocol developed by Philips and collaborators (Phillips et al., 2001). This protocol allows the obtention of the enriched extrasynaptic (Extra), postsynaptic (Post), and presynaptic (Pre) fractions from brain regions. In brief, the frozen tissue was thawed in cold buffer A (50 mM Tris-HCl pH 7.4, 0.32 M sucrose, 5 mM EDTA, 1 mM EGTA, 1 μg/ml aprotinin, 1 μg/ml leupeptin, 1/2500 PMSF, 20 μM ZnCl_2_, 50 mM NaF, 1 mM sodium orthovanadate, 2.5 mM sodium pyrophosphate, and a cocktail of protease inhibitors [Complete, Roche]) and mechanically homogenized using a potter. The homogenate was centrifuged for 10 min at 1,400 g, and the supernatant was collected. To increase the yield of protein recovery, this step was repeated thrice. The collected supernatants were pooled and centrifuged for 10 min at 700 g. The resulting supernatant was again centrifuged for 15 min at 21,000 g. The resultant pellet containing the crude membrane fraction was kept for further subfractionation, as follows. Crude membrane fraction was solubilized in buffer B (0.32 M sucrose, 50 mM Tris-HCl, pH 7.4) and loaded on a discontinuous sucrose step gradient (0.85 M/1.0 M/1.2 M). Further after centrifugation at 82,500 g for 2 h, the synaptosomes (Syns) were collected from the 1.0 M/1.2 M interface and diluted in 50 mM Tris-HCl pH 7.4 and centrifuged once more for additional 30 min at 21,000 g. Then this pellet was collected and resuspended in synaptosomal resuspension buffer, consisting of 36 mM sucrose 0.1 mM CaCl_2_ + 10 mM NaF + 1 mM sodium orthovanadate, 2 mM EDTA, protease, and phosphatase inhibitor cocktail (Halt^TM^, ThermoFisher), leading to the purified synaptosomal fraction (P2) for further subsynaptic fractionation, as follows. In brief, synaptosomes were solubilized by resuspending in 40 mM Tris-HCl pH 6.0 containing 1% triton X-100 and incubated at 4°C for 30 min under agitation. Following centrifugation at 40,000 g for 30 min, the supernatant fraction (corresponding to the extrasynaptic fraction) was acetone precipitated. The pellet, containing the synaptosomal junction, was resuspended in 20 mM Tris-HCl buffer pH 8.0 supplemented with 1% triton X-100. After 30-min incubation at 4°C, the fractions were separated by centrifugation at 40,000 g for 30 min. The resulting supernatant, corresponding to the enriched presynaptic fraction, was acetone precipitated. The pellet, containing the enriched postsynaptic fraction, was resuspended in 50 mM Tris buffer—2% SDS, as well as the resulting acetone-precipitated subsynaptic fractions.

For western blot analysis, equal protein amounts from each sample were loaded and separated using 8 and 10% SDS-PAGE. The proteins were transferred onto nitrocellulose membranes (Invitrogen) using iBlot semi-dry blotting system (Life Technologies). After transient staining of transferred proteins by Ponceau S staining, the membranes were blocked with 10% skim milk in TBST (10 mM Tris-HCl, 100 mM NaCl, 0.1% Tween-20) for 1 h. The membranes were incubated overnight at 4°C with the corresponding primary antibodies (diluted in TBST/+5% skimmed milk) and followed by incubation with HRP-conjugated anti-mouse, anti-guinea pig IgG-HRP, or anti-rabbit IgG secondary antibodies (Dako) for 1 h at room temperature. The detection was performed with chemiluminescence using Amersham ECL Prime Western blotting detection reagent (GE, Amersham) according to the manufacturers' instructions. The immunoreactive bands were visualized using a ChemiDoc MP (BioRad), and the immunoreactive signals were analyzed using the Image Lab Biorad software.

### Nano-UPLC mass spectrometry system and phosphopeptide enrichment

Mass spectrometry experiments were performed from two independent biological replicates. Each replicate consisted of a pool of hippocampi (*N* = 4 mice/genotype) that were subsequently fractionated to obtain subsynaptic fractions, either from euploid or from trisomic mice. Following resuspension and protein quantification, subsynaptic fractions (70–200 μg) were diluted in 1% SDS and digested with the single sequence-specific protease trypsin, following the previously described FASP protocol (Wiśniewski et al., 2009). Digested peptides were subjected to phosphopeptide enrichment using the High-Select™ TiO_2_ Phosphopeptide Enrichment Kit (Thermo Fisher Scientific). About 45% of each enriched sample was analyzed using an Orbitrap Fusion Lumos with an EASY-Spray nanosource coupled to a nano-UPLC system (EASY-nanoLC 1000 liquid chromatograph) equipped with a 50-cm C18 column (EASY-Spray; 75 μm id, PepMap RSLC C18, 2-μm particles, 45°C). Chromatographic gradients started at 5% buffer B with a flow rate of 300 nl/min and gradually increased to 22% buffer B in 79 min and to 32% in 11 min. After each analysis, the column was washed for 10 min with 95% buffer B (buffer A: 0.1% formic acid in water and buffer B: 0.1% formic acid in acetonitrile). The mass spectrometer was operated in data-dependent acquisition mode, with full MS scans over a mass range of *m*/*z* 350–1500 with detection in the Orbitrap (120-K resolution) and with auto gain control (AGC) set to 100,000. In each cycle of data-dependent acquisition analysis, following each survey scan, the most intense ions above a threshold ion count of 10,000 were selected for fragmentation with HCD at normalized collision energy of 28%. The number of selected precursor ions for fragmentation was determined by the “Top Speed” acquisition algorithm (maximum cycle time of 3 s), and a dynamic exclusion of 60 s was set. Fragment ion spectra were acquired in the ion trap with an AGC of 10,000 and a maximum injection time of 200 ms.

### Raw data processing and data analysis

Acquired data were analyzed using the Proteome Discoverer software suite (v2.0, Thermo Fisher Scientific), and the Mascot search engine (v2.5.1, Matrix Science) was used for peptide identification. Data were searched against a *Mus musculus* protein database derived from SwissProt and included the most common contaminants. A precursor ion mass tolerance of 7 ppm at the MS1 level was used, and up to three missed cleavages for trypsin were allowed. The fragment ion mass tolerance was set to 0.5 Da oxidation of methionine; N-terminal protein acetylation and phosphorylation in serine, threonine, and tyrosine were defined as variable modification; and carbamidomethylation of cysteines was set as fixed modification. The identified peptides were filtered by 5%FDR, and only those proteins identified in both biological replicates (for each condition) were retained for further analysis (Supplementary Table 1).

### Bioinformatic analysis of proteomic data

The proteomic signature of the experimental groups was represented using Venn diagrams. These diagrams were created using VENNY 2.1 freeware (http://bioinfogp.cnb.csic.es/tools/venny/; Oliveros et al., 2007–2015). This graphical interface allowed the representation of proteins overlapped among groups, together with the visualization of differentially expressed proteins in the different groups/fractions.

To functionally classify the differentially expressed proteins from the different groups (subsynaptic fraction and genotype), the data were submitted to the Database for Annotation, Visualization, and Integrated Discovery (DAVID: http://david.abcc.ncifcrf.gov/). This software, based on the use of gene ontology (GO), was used for grouping the differentially expressed proteins into biological processes, molecular functions, and cellular components.

### *In Silico* analysis of phosphoproteomic data

An updated database of murine kinases and phosphatases was generated before the analysis of the phosphoproteomic data. To this end, Uniprot database was used as the reference library, proteins were sorted (e.g., “kinase” and “phosphatase” criteria), and the protein list was further manually curated. This database was used to analyze potentially differentially expressed kinases and phosphatases between genotypes (Supplementary Table 2).

The analysis of the proteome and phosphoproteome signatures was performed using, as inclusion criteria, the identification of the proteins and phosphosites in both biological replicates. Furthermore, the conserved phosphosites were classified based on their qualitative expression in the different subsynaptic compartments, as well as in their relative expression levels. Regarding the latter, proteins and phosphosites detected in both fractions were classified based on the relative TS:EU ratio, resulting from the mean MS peak area (TS) (replicates 1 and 2)/mean MS peak area (EU) (replicates 1 and 2). Ratios < 0.75 or >1.5 were considered as downregulated and upregulated protein levels, respectively (see Supplementary Table 3).

Following phosphoproteomic profile identification of trisomic subsynaptic fractions, a systems biology approach was conducted to infer the potential kinases associated with the phosphoproteomic profile. To this end, the Human protein Reference Database (Hprd.org) was used, and the putative kinases involved in the phosphoprofile were identified. Finally, an interaction network analysis was performed using the Search Tool for the Retrieval of Interacting Genes/Proteins database (STRING; http://www.string-db.org/). This bioinformatic tool allowed the generation of protein–protein interaction networks, integrating the kinases/phosphatases and iGluR phosphoprofile of Ts65Dn subsynaptic fractions.

### Statistical analysis

Comparison of immunolabeling signal intensities between euploid and trisomic hippocampal fractions was evaluated using the GraphPad software. All datasets passed the Kolmogorov–Smirnov normality test and were analyzed applying a two-tailed unpaired *t*-test with Welch's correction. Graph bars represent the mean ± SEM for each group.

## Results

### Subsynaptic proteomic signature of the Ts65Dn mice hippocampi

**Figure 1:**
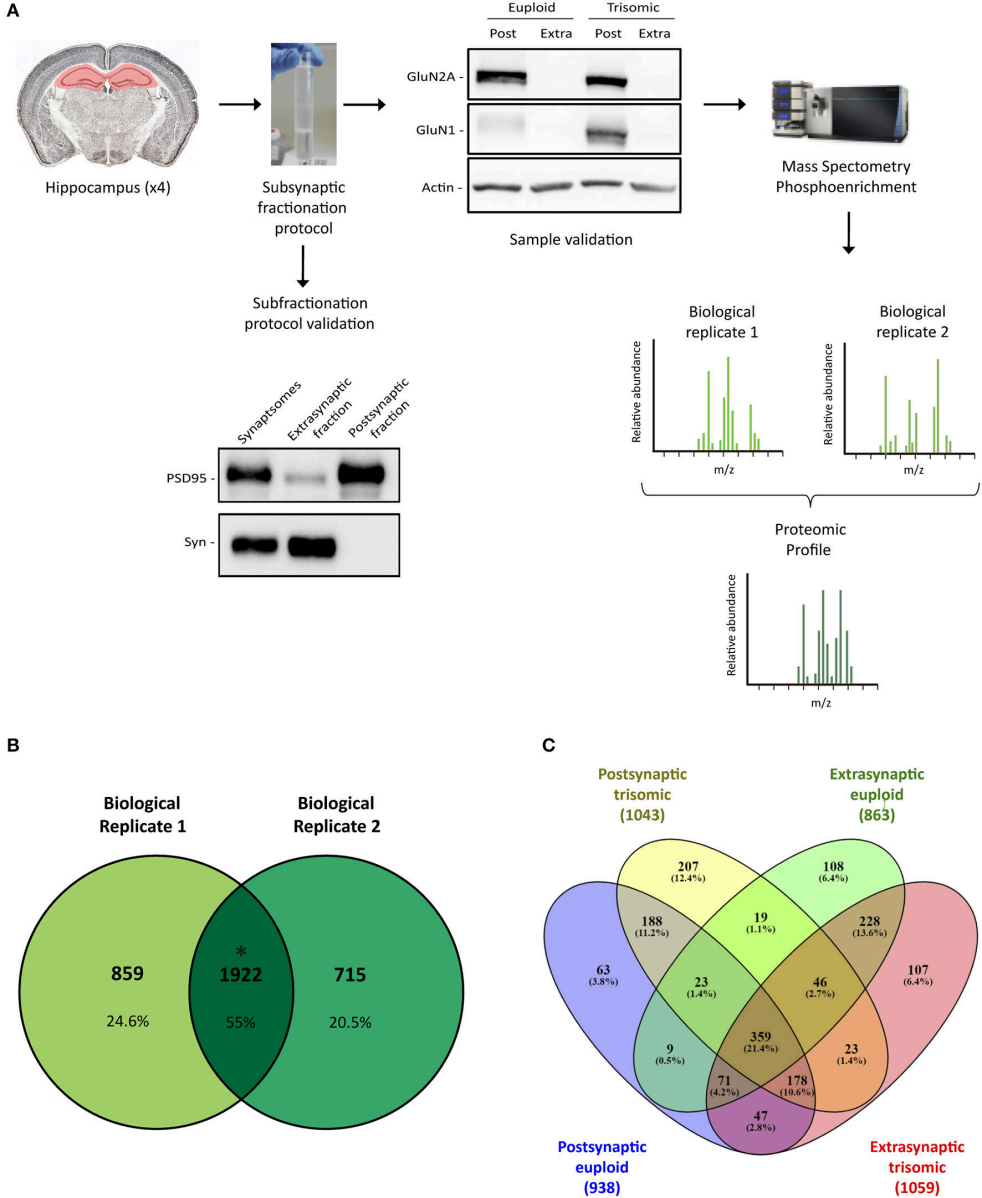
Subsynaptic fractionation and proteomic analysis of Ts65Dn mouse hippocampus. **(A)** Experimental workflow followed for hippocampal subsynaptic fractions obtention and mass spectrometry phosphoproteomic studies (mass spectra of biological replicates 1 and 2 are figurative representations). *Down left panel*, Representative western blot analysis of adult hippocampal subsynaptic fractions. Antibodies directed against PSD95 and synaptophysin were used as markers of postsynaptic and extrasynaptic, respectively. *Right up panel*, Western blot analysis of NMDAR subunits GluN2A and GluN1 in subsynaptic fractions (Post, postsynaptic; Extra, extrasynaptic) of adult euploid (EU) and trisomic (TS) mice. **(B)** Venn's diagram representing total identified proteins overlap between biological replicates 1 and 2 (postsynaptic and extrasynaptic fractions from both genotypes). **(C)** Venn's diagram representing genotype (EU, euploid; TS, trisomic) and subsynaptic compartment (Post, postsynaptic; Extra, extrasynaptic) effect on protein composition.

Neuronal communication mainly resides in the synapse, a highly specialized subcellular compartment juxtaposing crosstalking neurons. Within synapses, there is an additional morphological compartmentalization that allows the precise neurotransmitter and structural machinery positioning, underlying neuronal function. Consequently, potential specific changes on protein composition of the subsynaptic compartments can impact synaptic function and ultimately alter Ts65Dn glutamatergic neurons. To address this issue, we performed a subsynaptic fractionation protocol toward the obtention of extrasynaptic-, presynaptic-, and postsynaptic-enriched hippocampal fractions, both from euploid and from trisomic adult mice (Figure 1A).

Hippocampal subsynaptic fractionation protocol was validated by western blot, using specific subsynaptic compartment markers. As expected, extrasynaptic fraction showed an elevated signal of synaptophysin, whereas the postsynaptic fraction was enriched in scaffolding protein PSD95 (Figure 1A). Moreover, we observed a significant enrichment of the NMDA receptor subunits GluN1 and GluN2A in the postsynaptic fraction (Figure 1A). Overall, these biochemical studies confirmed the obtention of enriched subsynaptic compartments from murine brain tissue, and the same protocol was used to obtain enriched subsynaptic hippocampal fractions for mass spectrometry analysis.

Subsynaptic fractions (postsynaptic and extrasynaptic) were subjected to trypsin digestion, and mass spectrometry studies were performed. Data analysis was based on the comparison of the genotype effect on the subsynaptic proteomic/phosphoproteomic signature (defined as the overlapping profile of the biological replicates).

**Figure 2:**
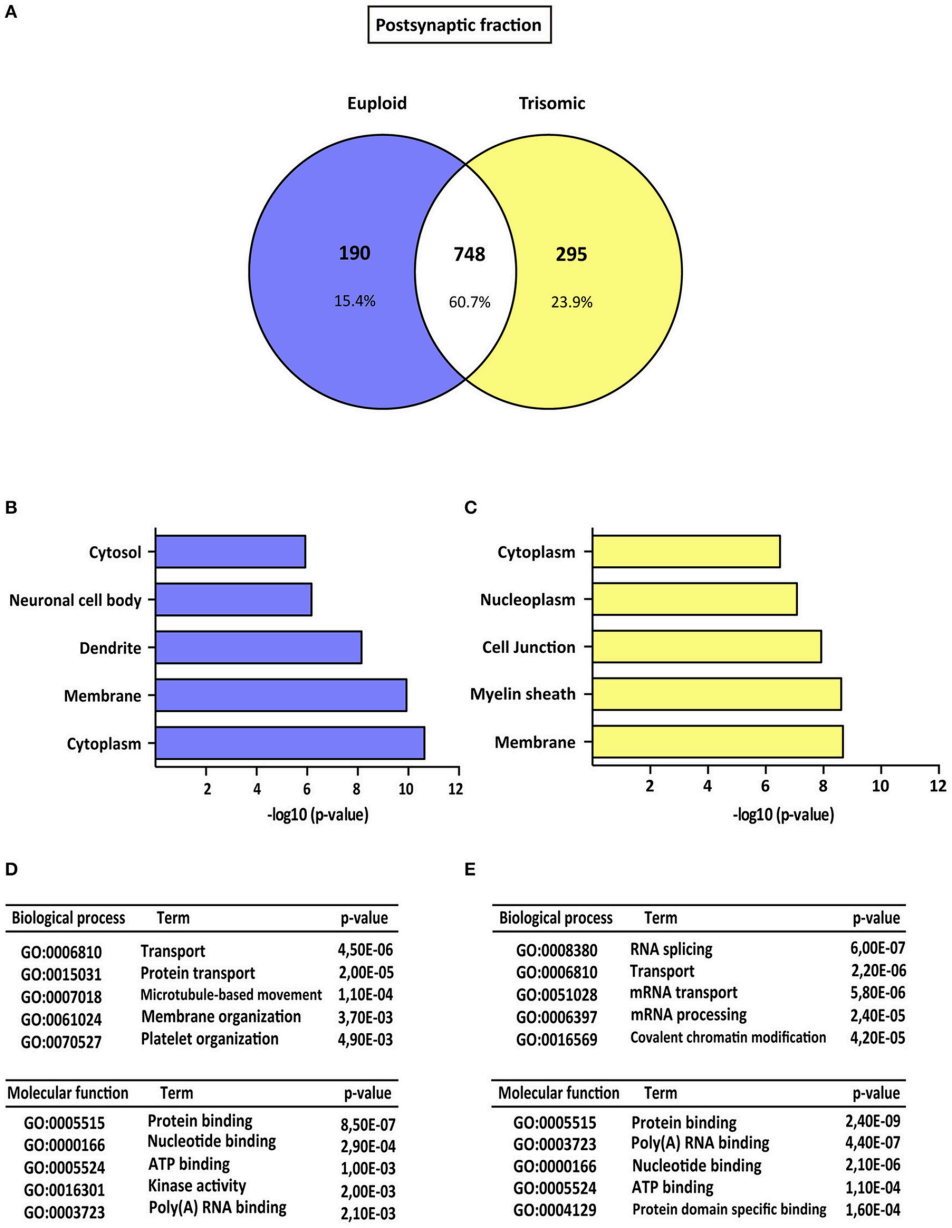
Gene ontology analysis of Ts65Dn mouse hippocampal postsynaptic proteome. **(A)** Venn's diagram representing comparative protein composition between postsynaptic fractions of adult euploid (EU) and Ts65Dn (TS) hippocampi. The diagram represents proteins detected in euploid replicates (blue circle), in trisomic replicates (yellow circle), and commonly detected in replicates from euploid and trisomic samples (white circle). **(B–E)** Functional annotation analysis of proteins enriched in hippocampal postsynaptic fractions of euploid **(B,D)** and trisomic **(C,E)** hippocampi **(B,C)**. Bar graphs representing top five significantly enriched GO terms, based on “cellular compartment” criteria **(D,E)**. Table listing top five significantly enriched proteins related with “biological process” and “molecular function” GO terms, in the postsynaptic fraction of euploid **(D)** and trisomic **(E)** hippocampi.

**Figure 3:**
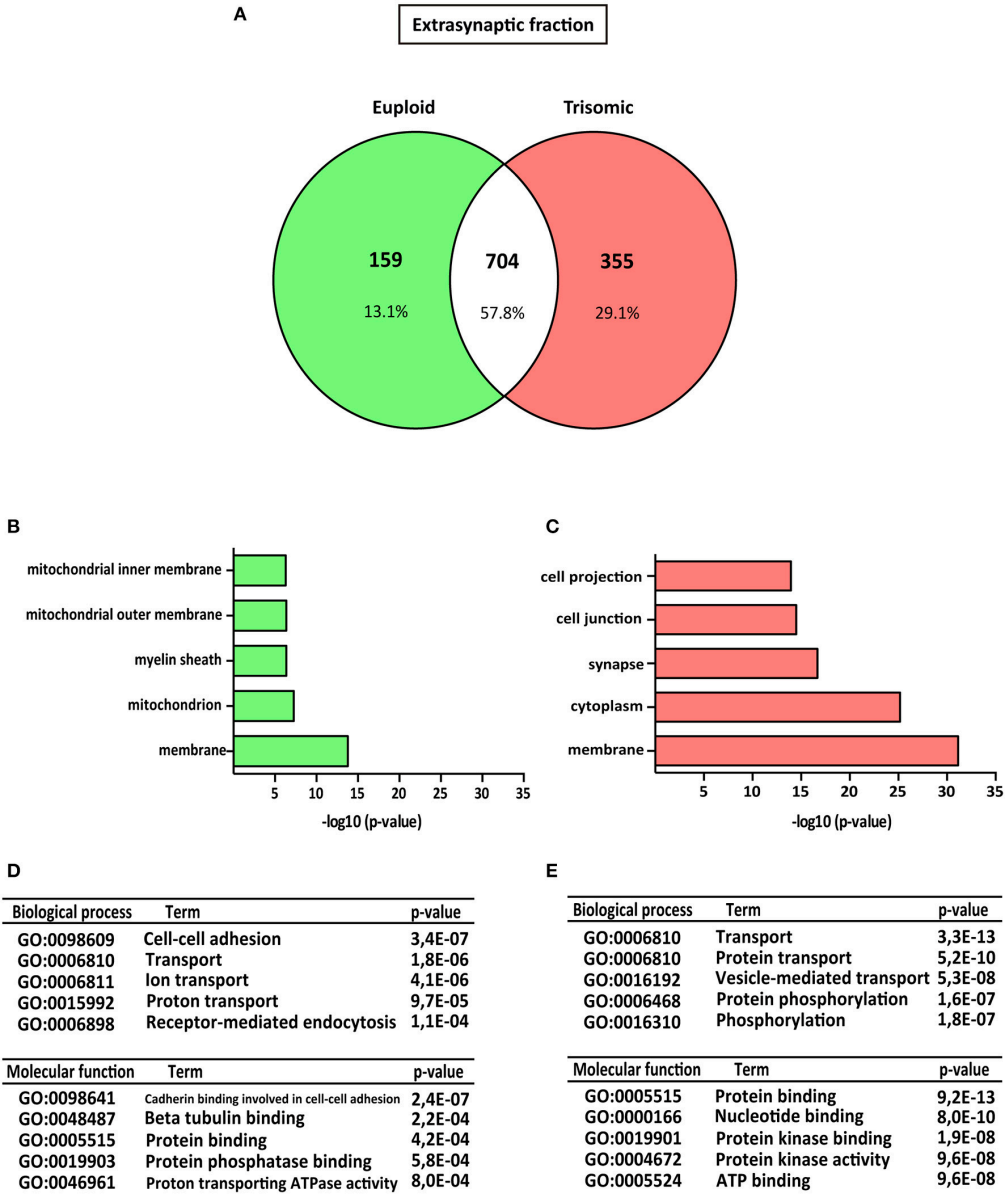
Gene ontology analysis of Ts65Dn mouse hippocampal extrasynaptic proteome. **(A)** Venn's diagram representing comparative protein composition between extrasynaptic fractions of adult euploid (EU) and Ts65Dn (TS) hippocampi. The diagram represents proteins detected in euploid replicates (green circle), in trisomic replicates (red circle), and commonly detected in replicates from euploid and trisomic samples (white circle). **(B–E)** Functional annotation analysis of proteins enriched in hippocampal extrasynaptic fractions of euploid **(B)** and trisomic **(C)** hippocampi **(B,C)**. Bar graphs representing top five significantly enriched GO terms, based on “cellular compartment” criteria **(D,E)**. Table listing top five significantly enriched proteins related with “biological process” and “molecular function” GO terms, in the extrasynaptic fraction of euploid **(D)** and trisomic **(E)** hippocampi.

Proteomic analysis allowed the identification of 1922 proteins (*M. musculus* protein database, SwissProt) consistently present in both biological replicates (Figure 1B). Subsynaptic distribution analysis of these proteins showed that 938/1043 proteins are present in the postsynaptic fraction of euploid/trisomic mice, respectively, whereas 863/1059 proteins are present in the extrasynaptic fractions of euploid/trisomic mice, respectively (Figure 1C and Supplementary Table 1). Despite the proteomic profile showed the presence of conserved proteins in trisomic hippocampal synapses (60.7 and 57.8% protein coincidence for postsynaptic and extrasynaptic fractions, respectively; Figures 2A, 3A), significant protein composition changes were detected in trisomic hippocampi. Further *in silico* analyses were performed to analyze the potential functional outcomes of these proteomic changes.

### Gene ontology enrichment analysis of subsynaptic Ts65Dn proteomic signature

The genotype effect on protein composition of the postsynaptic and extrasynaptic fractions was performed following the GO-term-based protein enrichment analysis. For functional annotation studies, only those proteins showing differential subsynaptic fraction expression were considered (Figures 2A, 3A). Statistical enrichment analysis of “cellular compartment,” “biological processes,” and “molecular functions” revealed the presence of significant genotype-dependent differences, both in the postsynaptic and in the extrasynaptic fractions (Figures 2B–E, 3B–E).

GO-term analysis of the euploid-specific postsynaptic proteome indicated the presence of an enriched representation of dendrite-associated proteins, thereby reflecting a dendritic protein under-representation in the trisomic postsynaptic fraction (Figures 2B–D). Moreover, GO analysis of the trisomic postsynaptic fraction showed significant changes in the following cellular functions: membranes, cytoplasm, transport, and protein binding (Figures 2C–E).

Regarding the extrasynaptic fractions, GO-term analysis of the euploid-specific extrasynaptic fraction showed the presence of mitochondria and protein phosphatase-binding proteins, indicating their relative absent detection in the trisomic extrasynaptic fraction (Figures 3B–D). In contrast, GO analysis of trisomic extrasynaptic fraction indicated changes in membrane, cytoplasm, synapse transport, phosphorylation, protein kinase activity, and protein binding (Figure 3C–E). Overall, GO-term analysis suggested that the subsynaptic proteome of the trisomic hippocampus is altered, in terms of both cellular compartments and functional outcomes. In particular, the analysis strongly suggested that phosphorylation-associated processes could be affected, as described below.

### Altered expression of kinases and phosphatases in the Ts65Dn mouse hippocampal subsynaptic fractions

**Figure 4:**
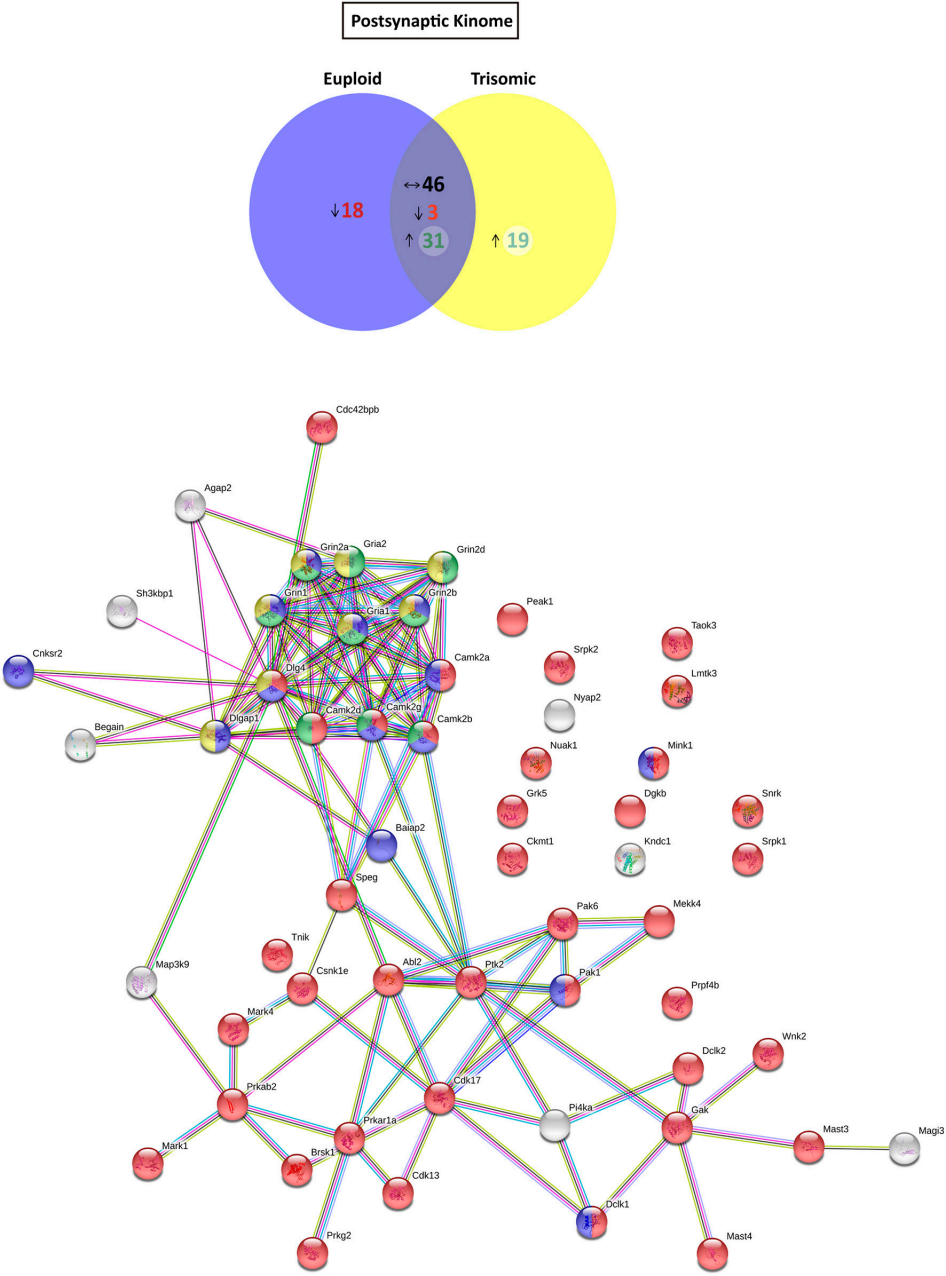
Kinome and kinase-iGluR interactome analysis of Ts65Dn mouse hippocampal postsynaptic fraction. **(Top)** Venn's diagram representing comparative analysis of protein kinases expressed in the postsynaptic fraction of adult euploid (EU) and Ts65Dn (TS) mice. The diagram represents proteins detected in euploid replicates (blue circle), in trisomic replicates (yellow circle), and commonly detected in replicates of euploid and trisomic samples (purple circle). The diagram shows the number of kinases not detected/underrepresented in Ts65Dn mice (in red), with similar expression (TS:EU expression ratio = 0.75–1.25; black characters) and present/overrepresented in trisomic mice (in green). **(Bottom)** Protein–protein interaction (PPI) network of protein kinases, scaffolding proteins, and iGluRs upregulated in Ts65Dn postsynaptic fraction. Node color annotation: red, kinase activity; blue, postsynaptic density-associated kinase; green, participation in long-term potentiation; and yellow, present in the glutamatergic synapse; link color annotation: known interactions (light blue: curated databases; pink: experimentally determined), predicted interactions (green: gene neighborhood; red: gene fusions; blue: gene co-occurrence), and others (light green: text mining; black: coexpression; purple: protein homology).

**Figure 5:**
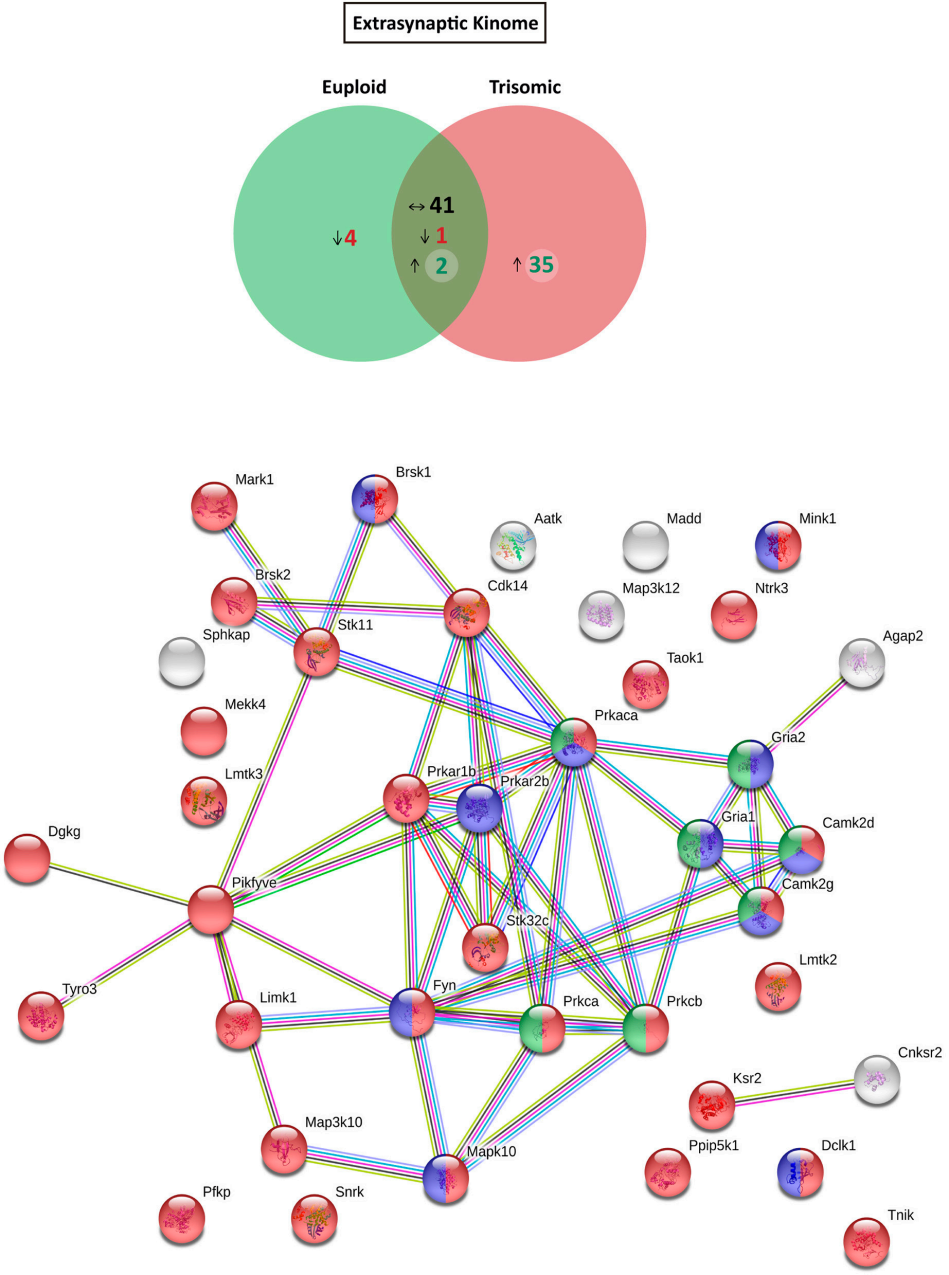
Kinome and interactome kinase-iGluR analysis of hippocampal extrasynaptic fraction of Ts65Dn mice. **(Top)** Venn's diagram representing comparative analysis of protein kinases expressed in the extrasynaptic fraction of adult euploid (EU) and Ts65Dn (TS) mice. The diagram represents proteins detected in euploid replicates (green circle), in trisomic replicates (red circle), and commonly detected in replicates of euploid and trisomic samples (dark green circle). The diagram shows the number of kinases not detected/underrepresented in Ts65Dn mice (in red), with similar expression (TS:EU expression ratio = 0.75–1.25; black characters) and present/overrepresented in trisomic mice (in green). (**Bottom)** Protein–protein interaction (PPI) network of protein kinases and iGluRs upregulated in Ts65Dn extrasynaptic fraction. Node color annotation: red, kinase activity; blue, postsynaptic density-associated kinase; green, participation in long-term potentiation; and yellow, present in the glutamatergic synapse; link color annotation: known interactions (light blue: curated databases; pink: experimentally determined), predicted interactions (green: gene neighborhood; red: gene fusions; blue: gene co-occurrence), and others (light green: text mining; black: coexpression; purple: protein homology).

Based on the functional annotation analyses showing alterations in phosphorylation, protein kinase activity, and phosphatase binding in the Ts65Dn synapses, we aimed to elucidate potential expression changes in kinases and phosphatases. To this end, an updated mouse kinome and phosphatome database was developed, using Uniprot as the reference database and filtering the *M. musculus* protein entries for the extraction of mouse kinases and phosphatases (Supplementary Table 2). Afterward, the genotype effect was compared either for postynaptic or for extrasynaptic fractions. With respect to euploid mice, the postsynaptic fraction of trisomic mice showed the presence of a subset of specific/enriched kinases and phosphatases (50 and 10 proteins, respectively), together with nondetectable/reduced expression of kinases and phosphatases (21 and 7 proteins, respectively) (Figure 4 and Supplementary Figure 1A). The comparative study of the extrasynaptic fractions indicated the presence, in trisomic samples, of a subset of specific/enriched kinases and phosphatases (37 and 7 proteins, respectively), together with non-detectable/reduced expression of kinases and phosphatases that are present in euploid samples (five and eight proteins, respectively) (Figure 5 and Supplementary Figure 1B). We performed western blot analysis of MS-identified kinases with putative differential expression to validate these unbiased findings. In agreement with proteomic data, western blot showed a significant increase of CamKII expression levels in the postsynaptic fraction of trisomic mice (*p* = 0.033, two-tailed Student's *t*-test), together with a significant decrease of Fyn kinase levels in the postsynaptic and a concomitant increase in the extrasynaptic fraction of the Ts65Dn mice (*p* = 0.015 and 0.024, respectively; two-tailed Student's *t*-test; Supplementary Figure 2A).

Further, we aimed to predict the potential consequences of the dysregulated trisomic synaptic kinome. We focused *in silico* analysis on the ionotropic family of glutamate receptors similar to the glutamatergic alterations reported in the Ts65Dn mouse model. The potential protein–protein interaction networks between iGluRs and protein kinases upregulated in the trisomic fractions (either postsynaptic or extrasynaptic) were analyzed using STRING software. Interactome analysis of the trisomic postsynaptic fraction unraveled significant kinase-iGluR interaction, as shown by the elevated number of edges (predicted number of edges: 123 vs. expected number of edges: 18) and the PPI enrichment (*p*-value = 0), indicating the significant interactions within the PPI network. In particular, STRING analysis identified protein kinases from the CaMKII family, Dlgap1, Agap2, MRCKβ distinctively interacting with the detected iGluR subunits (GluN1, GluN2A, GluN2B, GluN2D, GluA1, and GluA2; Figure 4). Interactome analysis of the trisomic extrasynaptic fraction unraveled significant kinase-iGluR interaction, as shown by the significant increase of the network edges (predicted number of edges: 51 vs. expected number of edges: 21) and the PPI enrichment (*p*-value = 2.49.10^−8^), indicating the significant interactions of the PPI network. Moreover, several kinases (CaMKII kinase family members, cAMP-dependent protein kinase, PKC family, and Agap2) distinctively interacting with GriA1 and GriA2 subunits of the AMPARs were also significantly detected (Figure 5).

### Altered iGluR subsynaptic phosphopattern in the Ts65Dn mouse hippocampus

**Figure 6:**
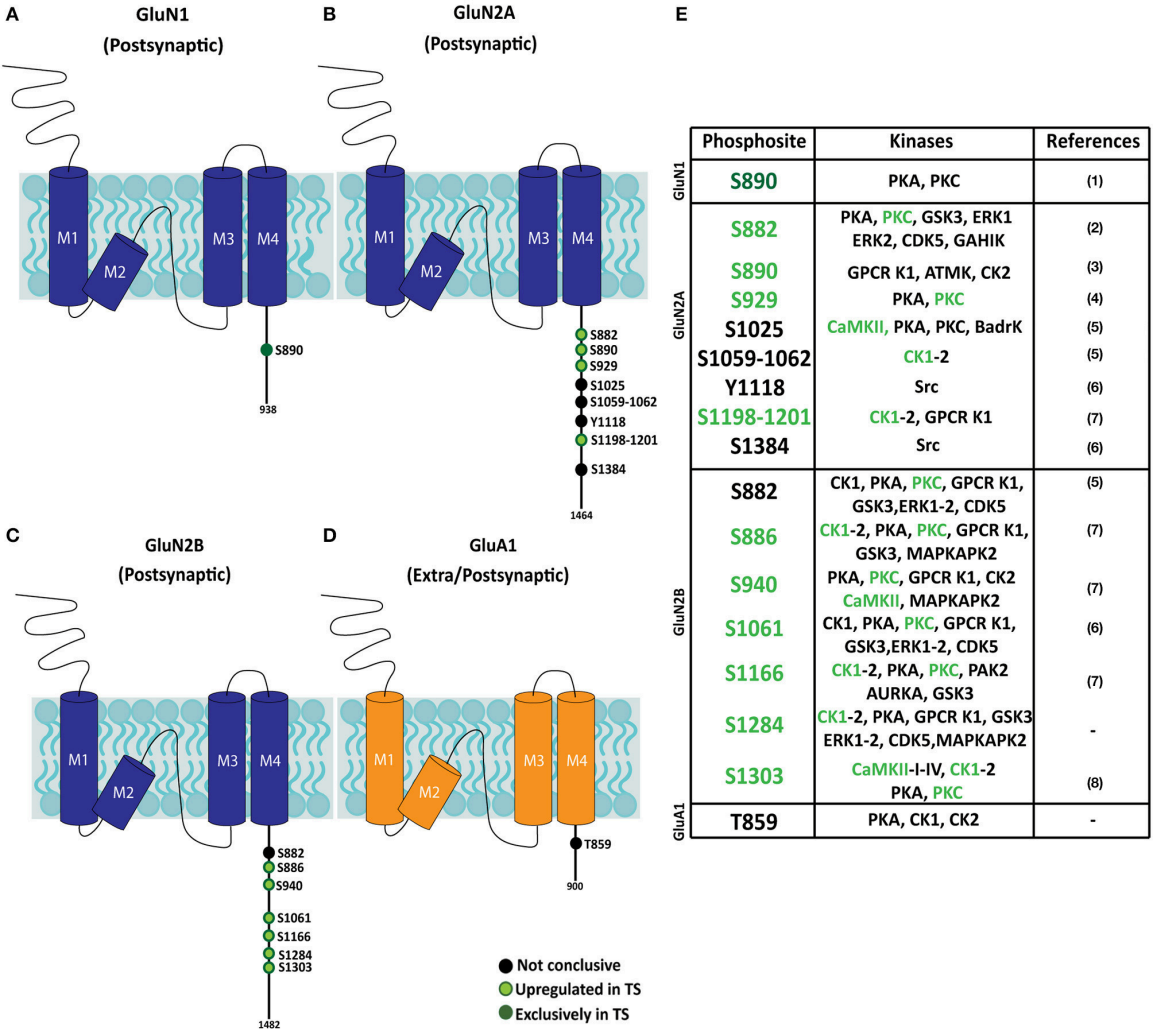
Phosphopattern profile of NMDA and AMPA receptor subunits in the adult Ts65Dn mouse hippocampus. **(A–D)** Schematic representation of phosphoproteome-identified phosphosites on NMDAR subunits (GluN1 and GluN2A and GluN2B) and AMPAR subunit GluA1, in adult hippocampal subsynaptic fractions. The phosphoresidue position is indicated, together with the presence and/or relative phosphorylation of trisomic:euploid MS peak (black: phosphosite present in euploid and trisomic samples; light green: increased levels detected in trisomic fractions; dark green: phosphosites exclusively detected in trisomic samples). **(E)** Table listing iGluR phosphosites identified in euploid and/or trisomic hippocampal fractions, together with the predicted phosphorylating kinases. Phosphosites previously reported in (1) Munton et al. (2007), (2) Lundby et al. (2012), (3) Li et al. (2016), (4) Maki et al. (2013), (5) Trinidad et al. (2012), (6) Zhang et al. (2013), (7) Wiśniewski et al. (2010) and (8) McQueen et al. (2017).

Hippocampal subsynaptic fractions were analyzed by phosphoenrichment-coupled mass spectrometry to evaluate the predictive kinome-iGluR interactome model. The phosphoproteome of the different fractions was analyzed, and a total of 59 iGluR phosphosites were identified in the different biological replicates (Supplementary Table 3). From these, only those iGluR phosphosites found in both replicates were retained for further genotype effect analysis (Figure 6 and Supplementary Table 3). This technique allowed the identification of several iGluR phosphosites, mostly corresponding to previously reported phosphoresidues (15 individual phosphosites); two novel phosphosites were identified. Importantly, the subsynaptic iGluR phosphopattern was characterized in the hippocampus of Ts65Dn mice. Using this unbiased method, the identified upregulated phosphosites (displaying higher MS peaks in trisomic samples) showed an elevated representation of NMDAR subunits (eight phosphosites for GluN2A, seven for GluN2B, and one for GluN1), whereas only one phosphosite was detected in GluA1 subunit (AMPAR subunit) and no phosphosites were identified in KAR subunits. We performed western blot analysis on selected MS-identified differentially expressed phosphosites to further validate our results. This analysis showed an increase of both GluN1(pS890) and GluN2B(pS1284) levels in the postsynaptic fraction of trisomic mice, although not significant, in agreement with the changes observed along unbiased phosphoproteomic analysis (Supplementary Figure 2B). Finally, the kinases putatively phosphorylating the consensus iGluR phosphoresidues were *in silico* predicted using “Phosphomotif Finder” software. This analysis predicted a potential enzymatic activity of CaMKII, PKC, CK1 on the identified iGluR phosphoresidues, which would be in agreement with their above mentioned upregulated expression.

## Discussion

In the present study, we have performed a phosphoenrichment-coupled mass spectrometry analysis that showed altered functional changes in the hippocampal extrasynaptic and postsynaptic fractions of the Ts65Dn mice, a trisomic mouse model recapitulating DS phenotypic alterations. The majority of kinases interacting with iGluRs remarkably show an overall increase in trisomic samples, whereas the majority of iGluR-related phosphatases show reduction, with some exceptions in both protein categories. These results are concomitant with the increased number of iGluR phosphopeptides identified by the phosphoproteomic analysis of Ts65Dn mice hippocampal postsynaptic fractions. Importantly, *in silico* kinome-iGluR phosphopattern analysis supported the role of specific kinases and their potential iGluR phosphosites in the neuronal pathophysiology of Ts65Dn mouse.

The initial proteome analysis showed a relative conservation of the number of proteins (roughly 1,000 proteins identified), regardless of the subsynaptic fraction and genotype. The comparative study of the genotype effect on the subsynaptic fraction composition showed a protein coincidence of 60.7 and 57.8% for the postsynaptic and extrasynaptic fractions, respectively. This quantitative analysis indicates a different molecular composition of the Ts65Dn hippocampal synapses, which might result from the overexpression of triplicated genes. Hence, several proteins are differentially expressed in the subsynaptic compartments of Ts65Dn hippocampal neurons, potentially reflecting the molecular substrates associated with Ts65Dn synaptic abnormalities. Interestingly, further GO-term-based protein enrichment analysis supported functional outcomes of the proteomic changes detected in the postsynaptic and extrasynaptic fractions of trisomic mice. In particular, the postsynaptic fraction of trisomic mice showed a relative decrease of proteins related with “dendritic function” GO term. This finding is in agreement with previous studies showing a reduction of dendritic branching and spine density in Ts65Dn mice (Belichenko et al., 2007) which, in the context of individuals with DS, might recapitulate the dendritic deterioration reported in DS fetal brains (Weitzdoerfer et al., 2001). Moreover, GO analysis revealed a differential enrichment of structural synaptic proteins within the extrasynaptic compartment. In particular, the trisomic extrasynaptic compartment showed a significant enrichment of GO-term “synapse.” Further analysis showed the presence of Homer3, one of the most abundant scaffolding proteins of the postsynaptic density in euploid mice (Hayashi et al., 2009), in extrasynaptic fractions of trisomic mice. These data, together with previous electron microscopy studies showing the presence of enlarged spines in Ts65Dn neurons (Belichenko, 2004; Benavides-Piccione et al., 2004), suggest that the morphological alterations of Ts65Dn spines might be associated with a disrupted protein composition of the extrasynaptic fraction. Interestingly, GO-term enriched analysis also showed a significant reduction of mitochondrial proteins in the extrasynaptic fraction of trisomic mice. This finding is in line with previous studies reporting a reduction of mitochondrial complexes and redox activity in neuronal cultures and DS fetuses, leading to severe mitochondrial dysfunctions that can affect synaptic metabolism, energy, and function (Izzo et al., 2014).

Besides the differential enrichment of structure- and energy-associated proteins, a significant alteration of phosphorylation-associated proteins was detected in the trisomic subsynaptic compartments. Indeed, the extrasynaptic trisomic fraction showed an enrichment of “protein phosphorylation” and “protein kinase activity” GO terms, while “protein phosphatase binding” (enriched in euploid extrasynaptic fraction) was significantly reduced. Although these evidences were suggestive of an altered enzymatic activity of kinases/phosphatases in Ts65Dn hippocampi, a more detailed analysis of kinase/phosphatase subsynaptic expression levels was performed. This analysis roughly showed an increase of kinase expression, together with a reduction of phosphatase levels in the Ts65Dn mouse model. In our study, further detailed analysis of the trisomic kinome showed increased levels of CaMKII subunits (alpha, beta, delta, and gamma subunits) in hippocampal postsynaptic and extrasynaptic fractions of Ts65Dn mice, by proteomics and WB techniques. This increase could result in an enhanced CamKII activity, similar to previous studies showing increased phosphorylated CaMKII in Ts65Dn hippocampi (Siarey et al., 2006). In agreement with this kinase increase, previous work of Fernandez and collaborators showed an increased amount of phosphopeptides in crude synaptosomes from Ts65Dn mice (Fernandez et al., 2009). Overall, our proteomic data and the previously cited morphological studies support the relationship between biochemical alterations and structural changes of the trisomic hippocampal neurons. These morphological and biochemical alterations can result in a subsynaptic mislocalization of proteins, potentially affecting synaptic shape, changing the postsynaptic density molecular composition, and ultimately the function synaptic activity of trisomic neurons. In summary, these results strengthen the hypothesis that morphological and functional dendritic/synaptic abnormalities, such as those detected in our study, may directly impact synaptic plasticity (Cramer and Galdzicki, 2012) and lead to cognitive impairment.

Interactome analysis revealed significant interactions between protein kinases enriched in trisomic subsynaptic fractions and iGluR subunits. In the postsynaptic fraction, these kinases interact with both NMDAR and AMPAR subunits. In contrast, the interactome analysis only revealed potential protein–protein interactions of these kinases with GluA1 and GluA2 subunits of the AMPAR, which are proportionally more largely represented than NMDAR subunits in the extrasynaptic fraction. Following this predictive analysis, we performed an accurate characterization of the iGluR phosphopattern in hippocampal subsynaptic fractions of Ts65Dn mice. Further bioinformatic analyses were focused on the phosphorylation of these receptors to unveil potential changes on iGluR phosphopattern. In sample preparation, the fractions followed tryptic digestion with a single protease (trypsin) for further unbiased MS method. Technically, the use of a single protease limited the extent of detectable phosphopeptides (Dephoure et al., 2013). Moreover, the reported low abundance of phosphopeptides (Parker et al., 2010) might also contribute to underestimate the complete phosphoproteome of trisomic hippocampal synapses. Despite these intrinsic technical limitations, a total amount of 17 individual iGluR phosphosites were identified in our study, 15 of them corresponding to previously reported phosphosites (PhosphositePlus, Cell Signaling Technology Inc.) (Lussier et al., 2015) (Figure 6E). In the past, Siarey and collaborators showed increased phosphorylation levels of iGluR phosphosites in Ts65Dn mice, such as GluA1(pS831) (Siarey et al., 2006). In line with these, our results showed an over-representation of iGluR phosphoresidues in specific Ts65Dn synaptic compartments identified, supporting the hypothesis that iGluR phosphopattern is associated with the synaptic transmission and glutamatergic dysfunctions reported in the Ts65Dn mouse model of DS. Furthermore, iGluR phosphoprofile analysis provided potential functional links between increased levels of iGluR sububnits in Ts65Dn synaptic fractions and *in silico* predicted kinases phosphorylating those phosphoresidues. This group of candidate kinases contained CaMKII, PKC, and CK1, previously described as iGluR regulatory kinases (Lan et al., 2001; Chergui, 2005; Sanz-Clemente et al., 2013; Lussier et al., 2015). Our proteomic study significantly showed an upregulation of these kinases in trisomic hippocampi, supporting the association between their overexpression and the changes on iGluR phosphopattern. Regarding the latter, a significant elevated representation of GluN2A and GluN2B subunit phosphopeptide was detected in trisomic postsynaptic fractions. This biochemical signature suggests a relevant contribution of abnormally phosphorylated GluN2A- and GluN2B-containing NMDARs in Ts65Dn phenotype. Indeed, our group and others have previously provided biochemical and pharmacological evidences supporting the role of NMDAR alterations in the DS murine models (Costa et al., 2007; Altafaj et al., 2008; Hanson et al., 2013; Grau et al., 2014). It has been reported that, specifically, CA3-CA1 hippocampal LTP is functionally reduced in Ts65Dn mice (Siarey et al., 2006). Moreover, trisomic mice exhibit an increased NMDAR-dependent LTD that can be pharmacologically rescued with memantine, an NMDAR antagonist (Scott-McKean and Costa, 2011). The alterations on NMDAR activity observed in Ts65Dn mice might probably be influenced by changes in NMDAR expression levels and/or the NMDAR (and other iGluR) phosphopattern signature described in the present study. Likewise, our previous studies showed that in the TgDyrk1A transgenic mouse model, DYRK1A overexpression is associated with GluN2A subunit upregulation (Altafaj et al., 2008). This increased density mechanistically results from a Dyrk1A-mediated phosphorylation of GluN2A subunit (GluN2A(pS1048)) that reduces NMDAR internalization and functionally increases NMDAR-mediated currents and modifies NMDAR gating properties (Grau et al., 2014). Unfortunately, the methodology followed in this study (trypsin digestion) was not compatible with GluN2A(pS1048) detection. Although our study revealed Dyrk1A presence in the hippocampal postsynaptic fraction and previous studies showed DYRK1A overexpression in DS, our data suggest that Dyrk1a-mediated phosphorylation GluN2A(pS1048) might contribute to DS synaptic alterations, a hypothesis that might be explored in the future. Interestingly, several iGluR phosphosites identified in this study have been associated with synaptic plasticity mechanisms. Among these, GluN1(pS890) has been showed to disturb GluN1 surface clusters (Tingley et al., 1997), and GluN2A(pS929) phosphorylation has been described as modulating GluN2A subunit containing NMDAR desensitization (Maki et al., 2013). Regarding GluN2B subunit phosphorylation, GluN2B(pS1303) phosphorylation has been shown to increase GluN1/GluN2B currents (Liao et al., 2001), and GluN2B(pS1116) phosphorylation potentiates NMDAR currents and Ca^2+^ permeability (Murphy et al., 2014). Overall, the presence of discrete NMDAR phosphosites with relevant function in synaptic function and the identification of their relative overrepresentation in trisomic mice support the contribution of glutamatergic neurotransmission alterations in Ts65Dn mice phenotype.

Furthermore, some of the phosphosites found to be over-represented in our study had previously been described to be involved in synaptic plasticity. For example, GluN1 S890 phosphorylation disperses the surface clusters of GluN1 (Tingley et al., 1997). GluN2A S929 phosphorylation modulates desensitization of GluN2A/NMDA receptors (Maki et al., 2013). GluN2B S1303 phosphorylation increases GluN1/GluN2B currents (Liao et al., 2001). GluN2B S1116 phosphorylation potentiates the receptor currents and its permeability to Ca^2+^ (Murphy et al., 2014). Consequently, their upregulation could lead to alterations in synaptic plasticity. Overall these findings suggest that the altered kinome and iGluR phosphopattern (mainly affecting GluN2A and GluN2B subunits) can contribute to the glutamatergic neurotransmission dysfunctions present in the Ts65Dn mouse model of DS.

In summary, our study revealed an altered subsynaptic composition of the adult Ts65Dn mouse hippocampus. These molecular subsynaptic alterations might functionally be underlying the Ts65Dn-associated dendritic deterioration, altered synapse organization, and mitochondrial dysfunction. Furthermore, this phosphoproteomic study provides an extensive repertoire of the iGluR phosphosites, together with the respective predicted kinases. Importantly, although the mechanistic studies remain elusive, the characterization of the iGluR phosphopattern and the predicted associated kinases in the Ts65Dn hippocampi may help to identify novel therapeutic targets for DS synaptopathy.

## Author contributions

XA and MG contributed to the conception and design of the study. MG performed the experimental and bioinformatic analyses, with the technical help from CG. MG, FC, and XA wrote the manuscript. All authors contributed to the revision of the manuscript revision and also read and approved the submitted version.

### Conflict of interest statement

The authors declare that the research was conducted in the absence of any commercial or financial relationships that could be construed as a potential conflict of interest.
