# Supplementary material for: Phosphoproteomic Alterations of Ionotropic Glutamate Receptors in the Hippocampus of the Ts65Dn Mouse Model of Down Syndrome
Source: Front Mol Neurosci. 2018 Jul 25;11:226. doi: 10.3389/fnmol.2018.00226 (PMC6095006; doi:10.3389/fnmol.2018.00226)
Supplement: Supplementary file 1 [file Presentation_1.PDF]

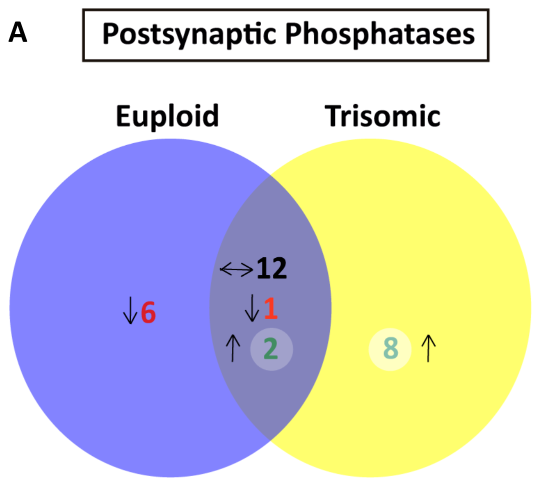

Supplementary Figure 1. Phosphatases profile in the Ts65Dn mouse model.
A. Venn diagram representing comparative analyses of protein phosphatases in the postsynaptic fraction of adult euploid (EU) and Ts65Dn (TS) mice. The diagram represents proteins detected in euploid replicates (blue circle), trisomic replicates (yellow circle) and commonly detected in replicates of euploid and trisomic samples (purple circle). The diagram shows the number of phosphatases not detected/underrepresented in Ts65Dn mice (in red), those equally represented (with a trisomic: euploid expression ratio between 0.75 and 1.25, black characters) and present/overrepresented in trisomic mice (in green). **B**. Venn diagram representing comparative analyses of protein phosphatases in the extrasynaptic fraction of adult euploid (EU) and Ts65Dn (TS) mice. The diagram represents proteins detected in euploid replicates (green circle), trisomic replicates (red circle) and commonly detected in replicates of euploid and trisomic samples (dark green circle). The diagram shows the number of phosphatases not detected/underrepresented in Ts65Dn mice (in red), those equally represented (with a trisomic: euploid expression ratio between 0.75 and 1.25, black characters) and present/overrepresented in trisomic mice (in green)
