# Supplementary material for: Phosphoproteomic Alterations of Ionotropic Glutamate Receptors in the Hippocampus of the Ts65Dn Mouse Model of Down Syndrome
Source: Front Mol Neurosci. 2018 Jul 25;11:226. doi: 10.3389/fnmol.2018.00226 (PMC6095006; doi:10.3389/fnmol.2018.00226)
Supplement: Supplementary file 2 [file Presentation_2.PDF]

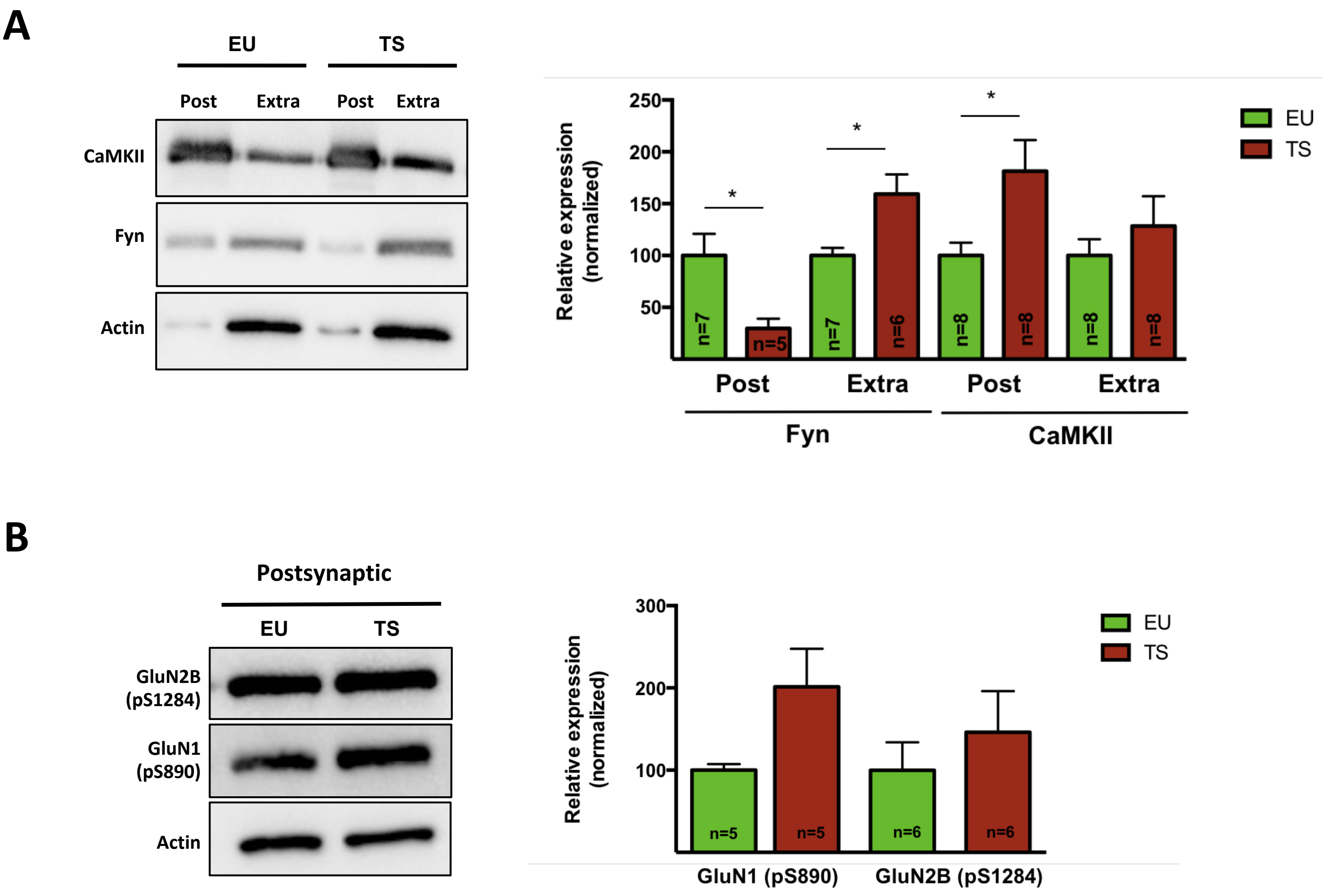


**Supplementary Figure 2. Comparative analysis of kinases and iGluRs phosphosites expression in euploid and trisomic Ts65Dn adult mice.**

**A.** Representative western blot analysis of postsynaptic and extrasynaptic fractions from euploid (EU) and trisomic (TS) hippocampi. Bar graph representing the relative average signal intensity ± SEM of CaMKII, Fyn and Actin (n = 5-8 mice / group). **B**. Representative western blot analysis of postsynaptic fractions from euploid (EU) and trisomic (TS) hippocampi. Bar graph representing the relative average signal intensity ± SEM of GluN1(pS890) (n = 5 mice / group) and GluN2B(pS1284) (n = 6 mice / group). *$p < 0.05$, two-tailed Student's *t*-test.
